# A combined approach for β-thalassemia based on gene therapy-mediated adult hemoglobin (HbA) production and fetal hemoglobin (HbF) induction

**DOI:** 10.1007/s00277-012-1430-5

**Published:** 2012-03-31

**Authors:** Cristina Zuccato, Laura Breda, Francesca Salvatori, Giulia Breveglieri, Sara Gardenghi, Nicoletta Bianchi, Eleonora Brognara, Ilaria Lampronti, Monica Borgatti, Stefano Rivella, Roberto Gambari

**Affiliations:** 1 Department of Biochemistry and Molecular Biology, Section of Molecular Biology, University of Ferrara, Ferrara, Italy; 2 Department of Pediatrics, Division of Hematology-Oncology, Children’s Cancer and Blood Foundation Laboratories, Weill Cornell Medical College, Cornell University, New York, NY 10021 USA

**Keywords:** β-thalassemia, Gene therapy, Lentiviral vectors, HbF induction, Erythroid progenitor cells

## Abstract

Gene therapy might fall short in achieving a complete reversion of the β-thalassemic phenotype due to current limitations in vector design and myeloablative regimen. Following gene transfer, all or a large proportion of erythroid cells might express suboptimal levels of β-globin, impairing the therapeutic potential of the treatment. Our aim was to evaluate whether, in absence of complete reversion of the β-globin phenotype upon gene transfer, it is possible to use fetal hemoglobin induction to eliminate the residual α-globin aggregates and achieve normal levels of hemoglobin. Transgenic K562 cell lines and erythroid precursor cells from β^0^39-thalassemia patients were employed. Gene therapy was performed with the lentiviral vector T9W. Induction of fetal hemoglobin was obtained using mithramycin. Levels of mRNA and hemoglobins were determined by qRT-PCR and HPLC. First, we analyzed the effect of mithramycin on K562 transgenic cell lines harboring different copies of a lentiviral vector carrying the human β-globin gene, showing that γ-globin mRNA expression and HbF production can be induced in the presence of high levels of β-globin gene expression and HbA accumulation. We then treated erythroid progenitor cells from β-thalassemic patients with T9W, which expresses the human β-globin gene and mithramycin separately or in combination. When transduction with our lentiviral vector is insufficient to completely eliminate the unpaired α-globin chains, combination of β-globin gene transfer therapy together with fetal hemoglobin induction might be very efficacious to remove the excess of α-globin proteins in thalassemic erythroid progenitor cells.

## Introduction

In β-thalassemias, mutations of the β-globin gene or its regulatory regions cause absence (β^0^ phenotype) or reduced synthesis (β^+^ phenotype) of β-globin chains [1–4], and this impairment leads to an excess of the complementary α-globin chains [1]. Ultimately, the precipitation of α-globin chains in excess promotes apoptosis of erythroid precursors in the bone marrow and at extra-medullary sites and shortens survival of red blood cells (BRCs) in the peripheral blood [5–10]. The disease is associated with morbidity and mortality due to severe chronic anemia or treatment-related complications [1].

Gene therapy is one of the possible approaches for the cure of β-thalassemia, following β-globin gene transfer in autologous hematopoietic stem cells (HSCs) [11–13]. Retroviral- or lentiviral-mediated insertion of single and multiple copies of the β-globin gene in ErPCs has been reported in many studies to demonstrate the feasibility of the gene therapy approach for the cure of β-thalassemia [11–17]. This approach, while straightforward in its principle, exhibits several critical issues, the major being the control of transgene expression, which needs to be: (a) erythroid-specific, (b) differentiation- and stage-restricted, (c) elevated, (d) position-independent, (f) sustained over time and (g) independent from the patient's genotype [18–21]. Moreover, the gene transfer, in order to be effective, needs to target the majority of HSCs, limiting the vector copy number to minimize the possibility of insertional mutagenesis. The study of Hargrove et al. [22] showed how lentiviral vector insertion can perturb the expression of endogenous genes in β-thalassemic hematopoietic cells [22]. Despite this evidence, clinical trials based on gene therapy on β-thalassemic patients have been initiated, although this therapeutic intervention was used on a restricted number of patients.

In the most recent study, a 19-year-old patient who received gene therapy treatment no longer needs palliative transfusions, and 2 years later, seems healthy. Still, the unexpected observation of the expansion of a group of clonal cells with the same gene insertion (the *HMGA2* gene) suggests that a lentiviral vector can promote growth advantage of selected cells [19]. The pattern is reminiscent of the gene therapy trial for the X-linked combined immunodeficiency disease (SCID) in which a retroviral vector triggered leukemia [23]. Therefore, the potential of gene therapy is limited by the number of viral particles that can be safely incorporated into the genome. In fact, the integration of fewer molecules is necessary to avoid genotoxicity, and at the same time, a minimum number of integration is required to sustain elevated transgene expression [15].

Our laboratory has been investigating the effects of several compounds on inducing the expression of γ-globin genes and the increase of fetal hemoglobin (HbF) synthesis [24–28]. It is generally believed that even small increases of HbF synthesis could be beneficial to β-thalassemia patients [29–31]. Although ErPCs from different patients might respond to a different extent to the same HbF inducer, our experience indicates that this effect is reproducible. The limitation of such approach is to reach levels of HbF clinically relevant. However, clinical trials with HbF inducers have been extensively investigated, using hydroxyurea (HU), thalidomide, and butyrates [32–36]. Following this research field, several studies focusing on the mechanisms regulating reactivation of HbF production in humans have been reported [27, 35, 37–39].

As for possible co-expression of γ-globin and β-globin genes, it should be considered that during ontogeny, two switches occur in β-like globin genes expression that reflect the changing oxygen requirements of the fetus, the second of which, from γ- to adult δ- and β-globin, occurs shortly after birth. Throughout the locus, *cis*-acting elements are dynamically bound by *trans*-acting proteins, including transcription factors, co-activators, repressors, and chromatin modifiers [40–42]. Despite the complex hierarchy of events regulating gene expression during development, from extracellular signaling to transcriptional activation or repression, the expression of β-globin and γ-globin genes appears to be inversely regulated (i.e., high expression of γ-globin genes versus low expression of β-globin genes and vice versa) [40, 41].

To our knowledge, no attempt has been made to verify whether induction of HbF and HbA might be obtained using cells isolated from homozygous patients unable to produce β-globin mRNA. This combined approach might have a synergistic effect, by adding HbA synthesis to high levels of HbF, which ameliorate the clinical parameters of β-thalassemia patients [28–31]. On the other hand, at least in theory, β-globin mRNA production following gene therapy approaches might interfere with γ-globin gene expression. The aim of this paper was to verify whether the HbF inducer mithramycin (MTH) stimulates the production of HbF in erythroid cells treated with a lentiviral vector carrying a therapeutic β-globin gene. The gene transfer vector utilized in this study (T9W) [17], is a third-generation lentivirus that has been obtained by modifying TNS9, a lentiviral vector of second generation. TNS9 was successfully utilized in mice to cure and rescue thalassemic mice affected by thalassemia intermedia and major, respectively [11, 14].

We analyzed the effect of MTH on K562 cell clones carrying the enhanced green fluorescent protein (eGFP) and the red fluorescent protein (RFP) driven by the γ-globin and β-globin promoter, respectively [43, 44]. We analyzed the effect of MTH on several transgenic K562 cell lines harboring different copies of the human β-globin gene to verify the possible co-expression of β-globin and γ-globin mRNAs, and the possible production of HbA and HbF under stimulation with MTH. K562 cells appear to be particularly useful in this context, since wild-type K562 cells do not express the endogenous β-globin gene, being exclusively committed to the expression of embryo-fetal globin genes [47, 48]. We then treated erythroid precursor cells (ErPCs) [49, 50] from β-thalassemic individuals with both MTH and the therapeutic T9W vector and analyzed globin mRNA expression by RT-PCR and production of HbF and HbA by HPLC.

## Materials and methods

### Lentiviral vector and chemical inducers

The T9W vector was generated by modifying TNS9, with the aim of increasing its safety and efficiency. For this purpose, the 3′ long terminal repeat (3′ LTR) was disarmed by deleting the U3 region (self inactivating-LTR or SIN-LTR) [17]. The deletion, which includes the TATA box and all the major determinants responsible for regulating the HIV-1 promoter, abolished the LTR promoter activity, but did not affect vector titers or transgene expression in vitro (data not shown). The *cis*-acting woodchuck post-regulatory element (WPRE) was also introduced [17]. Mithramycin (MTH) was purchased from Sigma (St.Louis, MO, USA) [23].

### Vector production and titration

Viral stocks were generated by co-transfection of the gene transfer plasmid T9W together with the envelope plasmid (VSV-G), the packaging plasmid (pMDLgpRRE), and the pRSV-REV plasmid into 293 T cells. An aliquot (5 x 10^6^) of 293 T cells was seeded into cell culture dishes (10 cm) 24 h prior to transfection in Iscove's modification of Eagle's medium (I-MEM, CAMBREX-Biowhittaker, Europe) with 10% fetal bovine serum (FBS, Biowest, Nuaillé, France) 100 U/ml penicillin, and 100 mg/ml streptomycin (Pen-Strep, CAMBREX-Biowhittaker, Europe), at 37°C under 5% CO_2_. The culture medium was changed 2 h prior to transfection. The precipitate was formed by adding the plasmids to a volume of 450 μl of 0.1× TE (0.1× TE is 10 mM Tris plus 1 mM EDTA) and 50 μl of 2 M CaCl_2_, then adding 500 μl of 2× HEPES-buffered saline (281 mM NaCl, 100 mM HEPES, 1.5 mM Na_2_HPO_4_) drop-wise, and then the precipitate was mixed and immediately added to the cultures. The medium (10 ml) was replaced after 16 h. Viral supernatant was collected at 24 and 48 h, cleared by low speed centrifugation, and filtered through cellulose acetate filters (0.2 μm). Following concentration by ultracentrifugation, serial dilution of concentrated virus (5; 0.5; and 0.05 μl, respectively) were used to infect 1 × 10^5^ NIH 3T3 cells in 1 ml of transfection buffer complemented with polybrene (Chemicon International, Millipore, Billerica, MA, USA) at a final concentration of 8 mg/ml. Genomic DNA was extracted after 3 days (Quiagen kit, Hilden, Germany). The multiplicity of infection (MOI) was calculated using the following formula: number of cells (1 × 10^5^) × dilution factor × VCN, measured via real-time PCR, using oligos for WPRE and ID genes (see below).

### Generation of K562 cell clones transduced with the pCCL.Promβ.HcRed1.Promγ.EGFP lentiviral vector

For determining the activity of the γ-globin and β-globin promoters under different treatment conditions, we modified the pCCL.PGK.GFP.WPRE construct, in which a constitutive expression of the eGFP gene is driven by the human phosphoglycerokinase gene promoter [43]. The PGK sequence was replaced by the γ-globin promoter to drive the expression of the eGFP gene. Additionally, we cloned into this construct the red fluorescent protein (RFP) gene together with the regulatory LCR and β-globin promoter elements. Human K562 cells [47] were used to obtain stable transfectants. In this system, increase of green eGFP signal will be consistent with a γ-globin gene promoter driven activity; on the contrary, increase of the far-red FP signal will be associated with β-globin promoter activity [43, 44]. For determining the promoter activity, cells were seeded at 12500 cells/ml and treated with MTH. After 5 days of culture, cells were assayed for fluorescent protein expression. For the determination of fluorescence intensity using the FACScan™ Flow Cytometer (Becton Dickinson, Franklin Lakes, NJ, USA), cells were harvested and washed; then, 10,000 cells were analyzed using the fl1 and the fl3 channels to detect green and red fluorescence, respectively, and analyses were carried out by using the Cell Quest (Becton Dickinson) software.

### Human K562 cell clones carrying the human β-globin gene

For the generation of stable K562 clones integrating human β-globin gene, the pCCL.β-globin.PGW vector was used [43, 44]. Transduction was carried out by plating 10^6^ K562 cells in 9.5-cm^2^ dishes with 45% RPMI and 45% I-MDM (Iscove's Modified Dulbecco's Medium, CAMBREX—Biowhittaker Europe), 10% FBS, 2 mM l-glutamine (CAMBREX—Biowhittaker Europe, Milan, Italy), 100 U/ml penicillin, and 100 mg/ml streptomycin in humified atmosphere of 5% CO_2_/air and adding the decided volume of the viral supernatant. In order to facilitate cell infection, 10 μl of the 800 μg/μl transduction agent polybrene (Chemicon International, Millipore, Billerica, MA, USA) was added to the K562 cells plated, which were subsequently cultured in a 5% CO_2_ incubator. After 7 days, cells were cloned by limiting dilutions and GFP-producing clones identified under a fluorescence microscope and further characterized. Cell cultures were maintained in humified atmosphere of 5% CO_2_/air in RPMI 1640 medium (SIGMA, St Louis, MO, USA) supplemented with 10% fetal bovine serum, 50 U/ml penicillin, and 50 mg/ml streptomycin. Cell growth was studied by with a ZF Coulter Counter (Coulter Electronics, Hialeah, FL, USA).

### In vitro culture of erythroid progenitors from β-thalassemia patients

Blood samples of patients were collected following receiving informed consent. The two-phase liquid culture procedure was employed as previously described [49, 50]. Mononuclear cells were isolated from peripheral blood samples by Ficoll–Hypaque density gradient centrifugation and seeded in α-minimal essential medium (α-MEM, SIGMA) supplemented with 10% FBS (Celbio, Milano, Italy), 1 μg/ml cyclosporine A (Sandoz, Basel, Switzerland), and 10% conditioned medium from the 5637 bladder carcinoma cell line. The cultures were incubated at 37°C, under an atmosphere of 5% CO_2_ in air, with extra humidity. After 7 days incubation in this phase I culture, the non-adherent cells were harvested, washed, and then cultured in fresh medium composed of α-MEM (SIGMA), 30% FBS (Celbio), 1% deionized bovine serum albumin (BSA, SIGMA), 10^-5^ M β-mercaptoethanol (SIGMA), 2 mM l-glutamine (SIGMA), 10^−6^ M dexamethasone (SIGMA), and 1 U/ml human recombinant erythropoietin (EPO) (Tebu-bio, Magenta, Milano, Italy) and stem cell factor (SCF, BioSource International, Camarillo, CA, USA). This part of the culture is referred to as phase II [47]. Erythroid differentiation was determined by counting benzidine positive cells after suspending the cells in a solution containing 0.2% benzidine in 0.5 M glacial acetic acid, 10% H_2_O_2_ [49]. Treatment with MTH was carried out by adding the appropriate drug concentrations at the beginning of the experiment (cells were usually seeded at 10^6^ cells/ml). For analysis of hemoglobins, cells were harvested, washed once with phosphate-buffered saline (PBS), and the pellets were processed in lysis buffer (0.01% sodium dodecyl sulphate). After spinning for 1 min in a microcentrifuge, the supernatant was collected and stored at 4°C.

### Transduction of erythroid precursors (ErPC) from β°-thalassemia patients with a lentiviral vector carrying the human β-globin gene

Mock control cells were compared to samples treated with MTH or T9W, separately. Moreover, an aliquot of the cells transduced with T9W were also treated with MTH. ErPCs were infected with serial dilutions of the virus, starting from multiplicity of infection (MOI) equal to 0.3 and multiples of it. ErPCs were routinely infected at the beginning of phase 2, when erythropoietin was administered to the cells to promote their erythroid commitment.

### RNA Isolation and RT-qPCR analysis

K562 clones and erythroid precursor cells were collected by centrifugation at 1,200 rpm for 5 min at 4°C, washed in PBS, lysed in 1 ml of TRIZOL® Reagent (GIBCO-Invitrogen-Life Technologies), according to the manufacturer's instructions. The isolated RNA was washed once with cold 75% ethanol, dried, and dissolved in diethylpyrocarbonate treated water before use. For gene expression analysis, 1 μg of total RNA was reverse transcribed by using random hexamers. Quantitative real-time PCR assay was carried out using gene-specific double fluorescently labeled probes in a 7700 Sequence Detection System version 1.7 (Applied Biosystems, Warrington Cheshire, UK) as described elsewhere [23, 24, 46]. The nucleotide sequences used for real-time PCR analysis are reported in Table 1. For real-time PCR analysis, we used as reference gene the endogenous control human GAPDH kit (Applied Biosystems). The fluorescent reporter and the quencher of the GAPDH probe were VIC and 6-carboxy-*N*,*N*,*N*′,*N*′-tetramethylrhodamine (TAMRA), respectively.

**Table 1:** Primers and probes for RT-qPCR

α-Globin	
Forward primer	5′-CACGCGCACAAGCTTCG-3′
Reverse primer	5′-AGGGTCACCAGCAGGCAGT-3′
Probe	5′-FAM-TGGACCCGGTCAACTTCAAGCTCCT-TAMRA-3′
β-Globin	
Forward primer	5′-CAAGAAAGTGCTCGGTGCCT-3′
Reverse primer	5′-GCAAAGGTGCCCTTGAGGT-3′
Probe	5′-FAM-TAGTGATGGCCTGGCTCACCTGGA-TAMRA-3′
γ-Globin	
Forward primer	5′-TGGCAAGAAGGTGCTGACTTC-3′
Reverse primer	5′-TCACTCAGCTGGGCAAAGC-3′
Probe	5′-FAM-TGGGAGATGCCATAAAGCACCTGC-TAMRA-3′

### High Performance Liquid Chromatography (HPLC)

Human erythroid precursor cells were harvested, washed once with PBS, and the pellets were lysed in lysis buffer (0.01% sodium dodecyl sulphate). After incubation on ice for 15 min, and centrifugation for 5 min at 14,000 rpm in a microcentrifuge, the supernatant was separated from the membrane debris and injected. Hb proteins present in the lysates were separated by cation-exchange HPLC [24], using a Beckman Coulter instrument System Gold 126 Solvent Module-166 Detector. Hemoglobins were separated using a Syncropak CCM 103/25 (250 × 4.6 mm) column, samples were eluted in a solvent gradient utilizing aqueous sodium acetate–BisTris–KCN buffers and detection was performed at 415 nm. The standard controls were the purified HbA (SIGMA, St Louis, MO, USA) and HbF (Alpha Wassermann, Milano, Italy) [23].

### Statistical analysis

The statistical significance of difference between treatments was analyzed, when appropriate, using one-way analysis of variance (ANOVA) and the Student–Newman Keul's test. *p* values lower than 0.01 were considered statistically significant.

## Results

### Co-existence of γ-globin and β-globin promoter activities in K562 cells

**Fig. 1:**
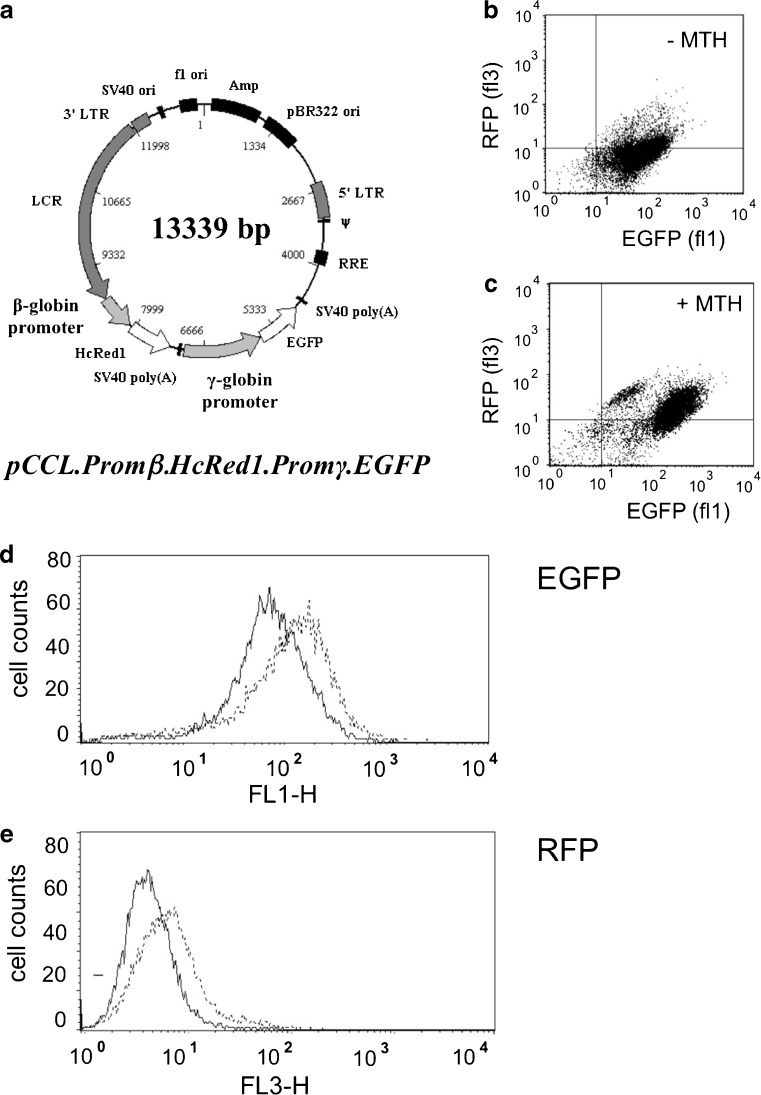
**a** Map of the pCCL.Prom*β*.HcRed1.Prom*γ*.EGFP vector, carrying the green fluorescence protein gene (EGFP) under the control of the *γ*-globin gene promoter and the red fluorescence protein gene (RFP) under the control of the β-globin gene promoter. **b**–**e** Representative effects of mithramycin on the expression of EGFP (**b**, **c**, **d**) and RFP (**b**, **c**, **e**). A stable clone of K562 cells harboring the pCCL.prom*β*.HcRed1.prom*γ*.EGFP vector was used and cultured in the absence (panel **b** and *solid lines* of panels **d** and **e**) or in the presence of 50 nM MTH (panel **c** and *dotted lines* of panels **d** and **e**). Analysis was performed after 5 days of MTH treatment

The first set of experiments was designed to determine whether co-existence of transcriptional activity driven by the human γ-globin and β-globin promoters might take place in human erythroid cells treated with MTH. To this aim, we used K562 cellular clones described in Guerrini et al. [43] and Lampronti et al. [44] and transduced the cells with the recombinant pCCL.Promβ.HcRed1.Promγ.EGFP vector (see Fig. 1a), carrying a green fluorescent protein (EGFP) and a red fluorescent protein (RFP) genes under the control of γ-globin and β-globin promoters, respectively [43, 44]. Using this experimental cellular system, the increase of green (eGFP) signal is consistent with a γ-globin promoter-driven transcriptional activity, while the increase of the far red (RFP) signal is associated with β-globin promoter activity [43].

In the experiment shown in Fig. 1b–e, K562 cells (12,500 cells/ml) were treated with 50 nM MTH and, after 5 days of culture, were assayed for fluorescent proteins expression. Figure 1c,d shows that MTH induces high levels of γ-globin promoter-driven transcription. This is expected due to the effect mediated by MTH on HbF induction and γ-globin gene transcription [23]. However, β-globin promoter driven transcription can also be activated by this concentration of MTH, as reported in panels c and e of Fig. 1. Treatment of K562 clones with 50 nM MTH induced a more modest but still net increase of RFP-associated fluorescence, compared to the eGFP-associate fluorescence. These data strongly suggest that both γ-globin and β-globin gene promoter activity can take place in MTH-induced K562 cells.

### Mithramycin treatment of K562 cell clones carrying multiple copies of the human β-globin gene

**Fig. 2:**
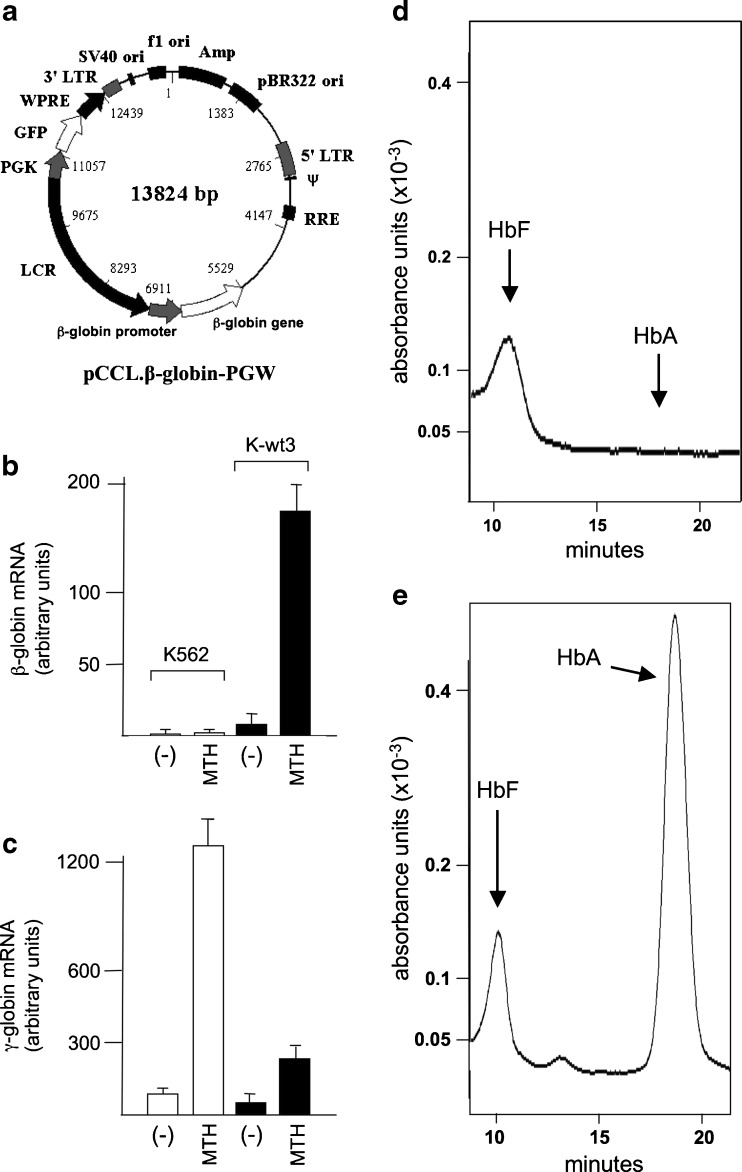
**a** Map of the pCCL.*β*-globin.PGW vector, carrying the human *β*-globin gene under the control of a mini-LCR as published elsewhere [45]. **b**, **c** Expression of β-globin (**b**) and γ-globin (**c**) genes following treatment of original K562 cells (*white boxes*) and K562-wt3 clone (*black boxes*) with 50 nM MTH. As evident only in the K562-wt3 clone, the *β*-globin gene is highly expressed. **d**, **e** Production of HbF and HbA following treatment of original K562 cells (**d**) and K562-wt3 clone (**e**) with 50 nM MTH. As evident, only in the K562-wt3 clone HbA is produced. Accumulation of globin mRNAs was analyzed by quantitative RT-PCR (for sequences of PCR primers see Table 1). Production of HbA and HbF was studied by HPLC. HbA and HbF were barely detectable in uninduced cells (data not shown)

Native K562 cells produce low amounts of hemoglobin and exhibit low proportion of benzidine-positive (hemoglobin-producing) cells at baseline [47, 48]. Following treatment with selected chemicals, K562 cells undergo erythroid differentiation exhibiting high levels of embryo-fetal hemoglobin (mainly Hb Gower-1 and Hb Portland). However, even in the erythroid-induced state, K562 cells do not exhibit β-globin mRNA expression [47]. We have previously shown that a lentiviral vector carrying this **β**-globin construct is able to express the β-globin mRNA and the corresponding protein in K562 cells after differentiation [45, 46]. This observation suggests that this combination of the genomic elements (gene, promoter, and LCR elements) from the β-globin locus is not recognized by the protein complex that suppresses β-globin transcription, at least in K562 cells. Therefore, K562 cells represent a suitable system to study activation of the transgenic β-globin gene harbored by pCCL.β-globin.PGW (see Fig. 2a). This construct was chosen for the expression of a GFP sequence, which greatly facilitates the identification of transduced cells, and the determination of the integrated copies of the gene. We have described elsewhere the infection of K562 cells with different MOI units of pCCL.β-globin.PGW vector and the isolation of ten clones that show, by real-time quantitative PCR analysis, different levels of genomic integration of the human β-globin gene [44]. We chose the K-wt3 clone which harbors one copy of the pCCL.β-globin.PGW vector/genome. Figure 2b, c shows the levels of β- and γ-globin mRNA expressions after MTH treatment of original K562 cells and of K-wt3 cells. High expression of β-globin mRNA was observed in MTH-induced clone K-wt3; on the contrary, very low expression of β-globin mRNA was detected, as expected, in original K562 cells, at baseline and after induction to erythroid differentiation by MTH. Both K562 and, to a lower extent, K-wt3 cells, accumulated γ-globin mRNA, which is further induced following treatment with MTH (Fig. 2c). Despite the fact that our experiments do not clarify the lower content of γ-globin mRNA in K-wt3 cells in respect to original K562 cells, the data obtained demonstrate that endogenous transcription of γ-globin mRNA can be carried out in the presence of de novo transcription of β-globin mRNA. In complete agreement, Fig. 2d, e shows that MTH-induced K562 cells produced HbF but not HbA (Fig. 2d), while MTH-induced clone K-wt3 synthetized both HbF and HbA (Fig. 2e), based on the HPLC analyses. High levels of embryo-fetal Hbs were observed in both original K562 cells and clone K-wt3 (data not shown). This indicates that, while original K562 cells are committed to preferential expression of embryo-fetal globins and hemoglobins, clone K-wt3, which carries a novel integrated unit of a functional human β-globin gene under the transcriptional control of this engineered LCR, is able to produce HbA in the presence of HbF induction.

### Treatment of erythroid precursor cells (ErPCs) from β-thalassemia patients with the T9W vector: effects on β-globin mRNA and HbA production

**Fig. 3:**
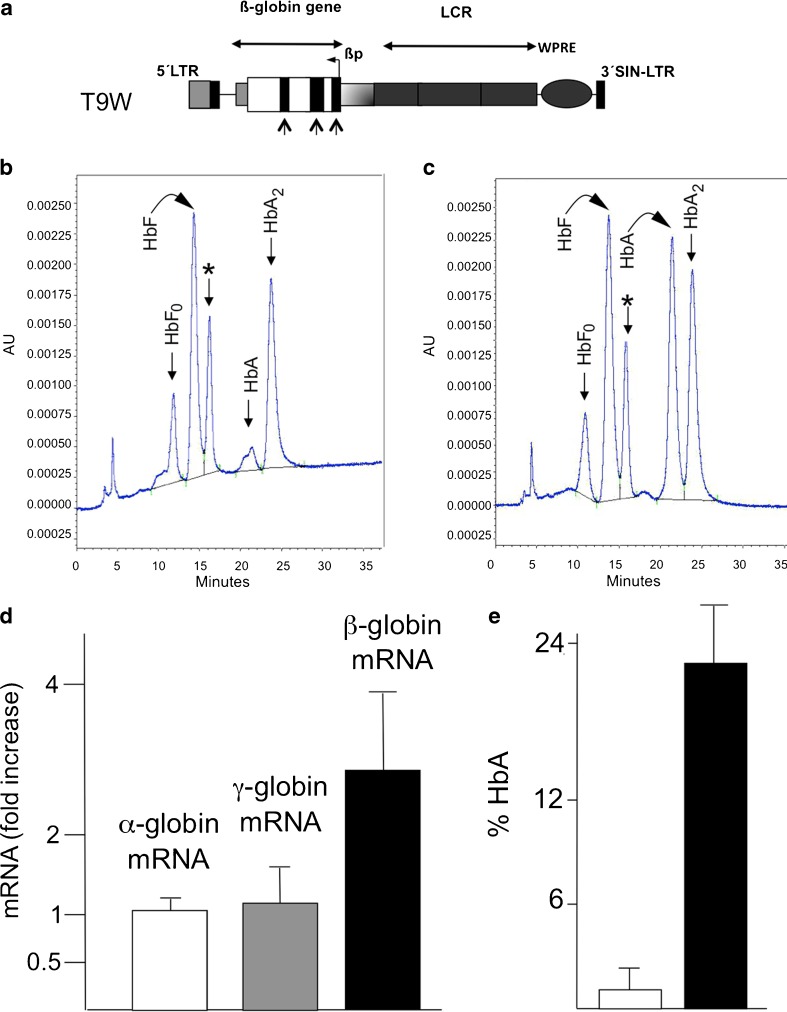
**a** The lentiviral vector utilized in this study (T9W), presenting the insertion of the CMV promoter in the 5′ LTR, a partial deletion of the 3′ LTR, and the inclusion of the woodchuck hepatitis virus posttranscriptional regulatory element (WPRE) to increase safety and production, respectively. *Black arrowed rectangles* represent exons of the β-globin gene [17]. **b**, **c** Ability of the employed lentiviral system (T9W) to induce accumulation of HbA following infection of erythroid precursor cells with T9W. A representative experiment is shown. **b** Uninfected ErPCs from a β^0^39-homozygous patient; **c** the same cells infected with T9W. An average integration of 0.95 copy/genome was obtained. Asterisks indicate the peaks corresponding to α-globin aggregates. **d** Fold increase of α-globin (*white box*), *γ*-globin (*gray box*), and β-globin (*black box*) mRNAs following T9W infection of ErPCs from seven β^0^39-homozygous patients. **e** HbA production in uninfected (*white box*) and T9W infected (*black box*) ErPCs. Data of panels **d** and **e** represent average ± SD from seven independent experiments

In order to have preliminary information on the efficacy to increase β-globin mRNA following treatment of β-thalassemic erythroid precursor cells with the therapeutic T9W vector (Fig. 3a) [17], ErPCs from seven homozygous **β** ^0^39-thalassemic patients were infected with T9W. At the end of the standard culture procedure, **β**-globin mRNA content was assayed by quantitative RT-PCR and HbA production by HPLC (Fig. 3b–e).

The results indicate that the ErPCs from these patients were responsive to gene transfer, as shown by the significant increase of β-globin mRNA content (Fig. 3d) without any changes in accumulation of α- and γ-globin mRNAs. Figure 3b, c shows a representative HPLC analysis of ErPCs lysates derived from a homozygous β^0^39-thalassemic patient at baseline (panel b) or after gene transfer with T9W (panel c). Noticeably, the HbA peak was detectable only in lysates of ErPCs that were infected with T9W (Fig. 3c). No major changes were observed on the production of HbF and HbA2. The relative high level of HbA2 is explained by the low overall protein levels of HbA and HbF proteins [17], whilst HbA2 is very low when HbF is expressed at very high levels, as indicated by HPLC analysis of transfusion-independent HPFH patients [51]. Interestingly, a decrease (but not complete suppression) of the peak corresponding to free α-globin (asterisk) was evident. These results confirm that T9W is able to convert β^0^39-thalassemic ErPCs (unable to produce HbA) to HbA-producing cells.

As expected, a direct correlation between MOI and HbA production was observed; moreover, decrease of the α-globin peak was achieved only when high MOI was employed. Unfortunately, at these MOI, multiple genomic integration of the T9W was obtained, an experimental condition that might produce unwanted expression of genes adjacent to integration sites.

### Treatment of T9W infected erythroid precursor cells from β^0^39-thalassemia patients with MTH: effects on production of globin mRNAs

In order to analyze the effects of MTH on transduced ErPCs, cells were infected with the T9W lentiviral vector alone or in combination with MTH, using MTH concentrations known to stimulate increased expression of the γ-globin genes without major changes in the **β**-globin mRNA production, as published by Fibach et al. [24] and reviewed in Gambari and Fibach [28]. We decided to perform this protocol using ErPC from β^0^39/β^0^39 thalassemic patients exhibiting different starting levels of HbF (ranging from about 10% up to 50%). Preliminary real-time RT-qPCR experiments performed on a large cohort of ErPCs normal donors and β^0^39/β^0^39 thalassemic patients allows us to conclude that (a) in thalassemic ErPCs the relative expression of α-globin genes is higher than that found in ErPCs from normal donors; (b) β-globin mRNA production is about 80% in respect to α-globin mRNA in ErPCs from normal donors, being not more than 2.5% in thalassemic ErPCs as expected, due to the nonsense mediated decay of the β^0^39-globin mRNA. The results of the quantitative RT-PCR data are shown in Table 2. As expected, based on the results that we previously reported [24], MTH has no effects on β-globin and α-globin gene expression (Table 2). However, MTH was able to stimulate γ-globin mRNA accumulation in erythroid progenitors, irrespectively from T9W transduction. In fact, in T9W transduced cells, increase of γ-globin mRNA production was induced by MTH together with de novo production of β-globin mRNA, which is exclusively associated with T9W transduction (Table 2). In addition, MTH had only minor effects on the amount of β-globin mRNA produced after infection with the T9W lentiviral vector. Furthermore, transduction with T9W had no effect on α-globin and γ-globin mRNA accumulation (Table 2).

**Table 2:** Accumulation of globin mRNA following treatment with MTH of T9W infected ErPCs from thalassemic patients

Genotype	Treatment	Globin mRNA increase		
		α-Globin mRNA	γ-Globin mRNA	β-Globin mRNA
β^0^39/β^0^39	MTH	1.02 ± 0.31	2.36 ± 0.55	0.95 ± 0.22
	T9W	1.15 ± 0.41	1.08 ± 0.35	2.32 ± 0.41
	T9W + MTH	1.03 ± 0.21	1.85 ± 0.28	2.12 ± 0.44

Data represent the fold changes (average ± S.D.) of the indicated mRNAs in respect to control untreated ErPCs. T9W was used at MOI generating an average of one integration/genome. MTH was used at 50 nM. Number of β^0^39/β^0^39 samples = 5

### Treatment of T9W infected erythroid precursor cells from thalassemia patients with MTH: effects on hemoglobin production

**Fig. 4:**
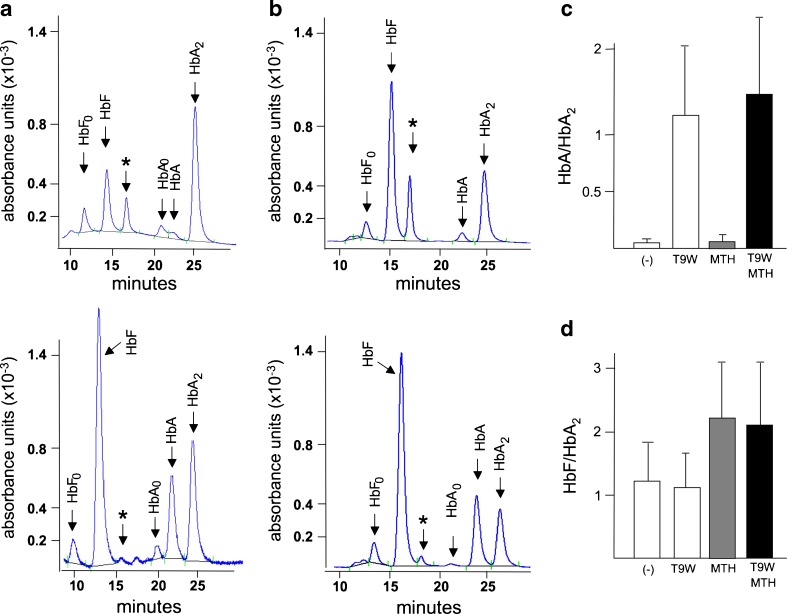
**a**, **b** Representative HPLC results on ErPCs from two β^0^39-homozygous patients infected with T9W in the presence of MTH (*bottom panels*); the *top panels* report HPLC profiles of untreated, uninfected ErPCs. Asterisks indicate the peaks corresponding to α-globin aggregates. **c**, **d** Summary of the effects of the different treatments indicated on ErPCs from five β^0^39-homozygous patients on HbA/HbA_2_ (**c**) and HbF/HbA_2_ (**d**) ratios. Results represent average ± SD (see data presented in Table 3). T9W was used at MOI generating an average of one integration/genome. MTH was used at 50 nM

Increased production of both γ- and β-globin mRNAs (Table 2) encouraged us to analyze by HPLC the production of hemoglobins. ErPCs of β^0^39/β^0^39 patients treated with both T9W and MTH exhibited increase of HbF and de novo synthesis of HbA (bottom part of the panels a and b of Fig. 4). No major effects (i.e., suppression or induction) on HbA were observed when erythroid progenitors were transduced with T9W alone or in combination with MTH (Table 3). HbF synthesis was always augmented in cells treated with MTH (with or without T9W), although in cells transduced with T9W, the MTH-induced increased HbF levels have to be considered in relationship with the simultaneous increased synthesis of HbA (Table 3). These data support the notion that induction of HbF by MTH and production of HbA with T9W might take place in the same ErPC cell cultures of β°-39-thalassemic patients; this conclusion is also reinforced by the analysis of the ratios HbF/HbA2 and HbA/HbA2 (Fig. 4c, d). When data from panels c and d of Fig. 4 were considered together with the results of Table 3, it appears evident that T9W transduced ErPCs from β^0^39/β^0^39 patients are induced to produce both HbA and HbF, when treated with MTH.

**Table 3:** Effects of treatment of ErPCs from homozygous β^0^39-thalassemic patients on hemoglobin production

Genotype	Hemoglobin	(−)	T9W	MTH	T9W + MTH
β^0^39/β^0^39	HbF	51.99	44.06	61.91	58.40
	α-peak	16.91	7.61	7.37	2.32
	HbA	3.29	24.01	2.9	21.45
	HbA_2_	27.64	24.27	27.80	17.79
β^0^39/β^0^39	HbF	32.43	29.93	51.05	43.31
	α-peak	35.37	30.65	24.19	19.15
	HbA	0.46	10.93	0.32	14.98
	HbA_2_	31.12	27.91	24.09	22.14
β^0^39/β^0^39	HbF	34.68	28.21	45.34	31.06
	α-peak	28.03	18.33	23.01	15.72
	HbA	0.63	23.05	0.44	25.15
	HbA_2_	36.35	29.85	30.33	27.56
β^0^39/β^0^39	HbF	25.19	19.91	60.01	53.13
	α-peak	11.31	5.78	6.12	2.37
	HbA	3.64	24.42	2.65	17.42
	HbA_2_	59.70	49.33	30.45	26.96
β^0^39/β^0^39	HbF	48.41	31.74	67.77	40.44
	α-peak	25.91	8.26	14.82	5.91
	HbA	1.05	44.71	1.84	41.47
	HbA_2_	23.99	14.93	15.39	11.79

Data represent the percent of the indicated hemoglobins (HbF, HbA, and HbA_2_). The α peak has been identified as a HPLC peak containing α-globin. T9W was used at MOI generating an average of one integration/genome. MTH was used at 50 nM

### ErPCs transduced with T9W and treated with MTH: amelioration of the phenotype

**Fig. 5:**
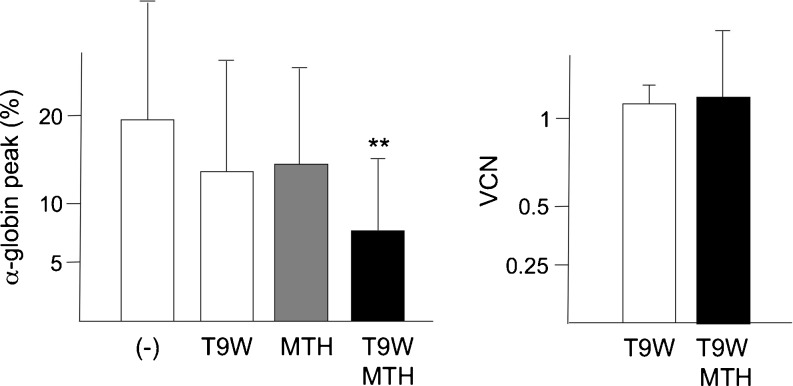
**a** Effects of treatment with T9W, MTH, and T9W + MTH on the α-globin peak content [17], shown in Figs. 3 and 4 with asterisks. *p < 0.05; **p < 0.01. T9W was used at MOI generating an average of one integration/genome. MTH was used at 50 nM. **b** VCN obtained in the five experiments performed and reported in Table 3

In all the HPLC analysis of ErPC cultures from thalassemic patients, in addition to HbF, HbA, and HbA2, a peak corresponding to α-globin chain aggregates (indicated by the asterisks in Figs 3 and 4) was observed. In consideration of the relationship between excess of α-globin chain content and pathophysiology of thalassemia, a reduction of this peak might be considered an important indication of the amelioration of the phenotype [15, 17]. In this respect, we like to point out that the combined treatment with T9W and MTH reduces dramatically the presence of the HPLC peak corresponding to α-globin chain aggregates as reported in Table 3 and in the summary data included in panel a of Fig. 5. Therefore, while the T9W transduction or the MTH treatment reduced the peak corresponding to the α-globin chain, the combined approach led to an additive and very efficient inhibitory effect on the production of α-globin aggregates. This was observed without exception in all the experiments performed (Table 3). This important effect was achieved using amount of T9W leading to ∼1 copy of the vector per genome (Fig. 5b) and MTH concentrations that did not have any effect on β-globin mRNA accumulation (Table 2) and HbA synthesis (Table 3).

## Discussion

The aim of the present investigation was to verify whether a co-treatment of ErPCs from β-thalassemia patients with lentiviral-mediated gene transfer and with inducers of fetal hemoglobin could be effective to stimulate HbF and HbA production and abolish the excess of α-globin accumulation in erythroid cells. To address this question, we employed the T9W lentiviral vector for the infection and mithramycin as HbF inducer. We previously reported the efficacy of these tools for gene therapy purposes [17] or HbF induction [23]. In this study, all thalassemic specimens were obtained from homozygous β^0^39-thalassemia patients.

The first set of results demonstrated that the T9W lentiviral vector was able to induce β-globin gene expression and protein synthesis in ErPCs from homozygous β^0^39-thalassemia patients, confirming observations previously reported [17]. As depicted in Fig. 3, this result was consistently reproduced. However, after infection, all samples continue to synthesize some, although reduced, free α-globin chains, indicating that the excess of α-globin content was not completely abolished.

The major finding of this manuscript is the formal demonstration that forced expression of β-globin and γ-globin genes, respectively, by gene transfer and HbF induction, profoundly improves hemoglobin synthesis among β-thalassemic ErPC cells. This is achieved by reducing or eliminating free α-globin chain aggregates, indicating that this approach can correct clinically relevant parameters in treated cells. In this respect, the restoration of a balance between α-globin and β-like globin chains (here γ-globins and β-globins) is associated with clear amelioration of the phenotype of thalassemic cells [52–54]. In this paper, we focused on the effects of co-treatment with MTH and the T9W lentiviral vector on cells derived from homozygous β^0^39-thalassemia patients, and similar findings were observed in preliminary experiments performed using ErPCs from two β^+^39-thalassemia patients carrying a β^0^39/β^+^IVSI-110 genotype (Zuccato et al., unpublished observations).

We believe that these data emphasize the clinical relevance of combining gene therapy with HbF induction for the cure of β-thalassemia. An elevated expression of fetal hemoglobin is beneficial to patients affected by thalassemia intermedia [28–34]. Several experiments conducted in animal models [55] as well as in patients treated with HbF inducers, support the use of HbF induction [28–31, 33]. Recently, Ehsani et al. [33] showed that a 6-month treatment of 16 transfusion-independent thalassemia intermedia patients with a 20 mg/kg/day dose of HU 4 days per week produced a significant increase of HbF resulting in the amelioration of hematological parameters. While a larger sample size study is needed to validate our data, these results appear to be well in agreement with independent reports showing a dramatic response of several β-thalassemia patients to HU-mediated induction of HbF [28, 32–34]. Relevant to the issues covered in this paper, Musallam et al. [56] analyzed the association between HbF levels and morbidity in β-thalassemia intermedia on a cohort of 63 untransfused patients who had also never received HbF induction therapy. There was a strong negative correlation between the HbF level and the total number of morbidities [56].

Our results suggest that the combination of gene therapy with HbF induction (GT/HbF strategy) might be very useful to eliminate the excess of α-globins detected by the HPLC analyses of ErPCs from β-thalassemia patients. This appears to be a major goal in therapeutic intervention on β-thalassemic erythroid cells and, if reached, is expected to ameliorate the physiological parameter of treated cells.

In conclusion, our data suggest that the GT/HbF strategy, employing the co-treatment of target erythroid precursor cells with a lentiviral vector carrying a therapeutic β-globin gene and the HbF inducer mithramycin, leads to forced de novo accumulation of HbA and increased production of HbF, ultimately suppressing the excess of free α-globin chains. These results might be relevant for establishing a protocol maximizing the production of clinically therapeutic hemoglobins in thalassemic ErPCs.

In addition, our findings strongly support the need of further studies employing co-treatment with gene-therapy lentiviral vectors and other fetal hemoglobin inducers (including DNA-based HbF inducers). These studies are crucial since mithramycin is a chemotherapeutic agent which might cause toxicity if used in life-long treatments. However, less toxic mithramycin analogs have been recently described which exhibit better pharmacokinetics and tolerance [57]. Finally, the results here presented support the use of vectors carrying the β-globin gene together with sequences enabling the production of HbF. Recently, Wilber et al. [58] showed that a lentiviral vector encoding a short-hairpin RNA targeting the γ-globin gene repressor BCL11A was able to increase HbF levels from 33% to 45% in β-thalassemic erythroid cells, without compromising erythroid differentiation. On the basis of these findings and on the results here presented, novel vectors carrying, in addition to the therapeutic β-globin gene, sequences driving the production of shRNAs targeting mRNA encoding a repressor of human γ-globin gene transcription would be of interest, since they are expected to force β-globin gene transcription together with reactivation of γ-globin genes and HbF production.

## Abbreviations

HbA: Adult hemoglobin
HbF: Fetal hemoglobin
MTH: Mithramycin
ErPC: Erythroid precursor cell
HSC: Hematopoietic stem cell
HU: Hydroxyurea
VCN: Vector copy number
MOI: Multiplicity of infection
PBS: Phosphate-buffered saline
eGFP: Enhanced green fluorescent protein
RFP: Red fluorescent protein
FACS: Fluorescence-activated cell sorting
LTR: Long terminal repeat
LCR: Locus control region
WPRE: Woodchuck post-regulatory element
RBC: Red blood cell
